# Physical activity status prevents symptoms of long covid: Sulcovid-19 survey

**DOI:** 10.1186/s13102-023-00782-5

**Published:** 2023-12-14

**Authors:** Juliana Quadros Santos Rocha, Eduardo Lucia Caputo, Yohana Pereira Vieira, Max dos Santos Afonso, Suele Manjourany Silva Duro, Mirelle de Oliveira Saes

**Affiliations:** 1 Federal University of Rio Grande, Visconde de Paranaguá, 102, Centro, Rio Grande, Rio Grande do Sul 96203-900 Brazil; 2 Brown University, Providence, RI 02912 USA; 3 Federal University of Pelotas, Gomes Carneiro, 01, Balsa, Pelotas, Rio Grande do Sul 96010-610 Brazil

**Keywords:** Exercise, Long COVID, Cross-sectional studies, COVID-19

## Abstract

**Background:**

Physical activity is indicated as a treatment for Long COVID, but prevention is unknown. This study aimed to investigate the relationship between physical activity (PA) before and after acute SARS-Cov-2 infection and the presence of Long COVID symptoms in adults.

**Methods:**

We used data from the Sulcovid-19, a longitudinal study carried out with individuals who were infected by SARS-Cov-2 between December/2020 and March/2021. Participants were asked about 19 symptoms commonly associated with long COVID. Three PA variables were built, as follows: (1) remained inactive; (2) became inactive; (3) remained active.

**Results:**

2.919 people were interviewed. The prevalence of individuals who had at least one symptom of Long COVID is 48.3% (95%CI 46.5–51.1). Our results showed that 71.8% (95%CI 70.1–73.4) of the individuals remained inactive, 14.9% (95%CI 13.6–16.2) became inactive and 13.3% (95% CI 12.1–14.6) remained active. The likelihood of experiencing long COVID symptoms was reduced in the musculoskeletal (PR 0.70; 95%CI 0.49–0.99), neurological (PR 0.61; 95%CI 0.43–0.88), and respiratory (PR 0.58; 95%CI 0.35–0.96) systems in those who remained active. In addition, the likelihood of experiencing Long COVID symptoms was 7% less in those who remained active.

**Conclusions:**

Continuous PA practice showed important protection effect for Long COVID symptoms in adults.

**Supplementary Information:**

The online version contains supplementary material available at 10.1186/s13102-023-00782-5.

## Introduction

The societal, economic, and public health impacts of the COVID-19 pandemic have been extensive [1]. Since the pandemic was declared until January 2023, there have been more than 657 million reported cases of COVID-19 worldwide, resulting in over 6.6 million deaths. In Brazil the number of cases has exceeded 36.4 million, with over 694 thousand deaths [2].

Long COVID refers to the persistence of symptoms for a minimum of three months, lasting at least two months without an alternative diagnosis [3, 4].This condition can affect individuals who experienced mild or severe forms of the disease [5]. Among non-hospitalized individuals, the prevalence of Long COVID can reach as high as 34.0% [6].

Physical activity (PA) is widely recognized as an effective non-pharmacological approach to prevent and treat several chronic physical and mental diseases [7]. Previous research suggested an association between physical inactivity and COVID-19, demonstrating that physically active individuals have a 20.0–26.0% reduced likelihood of testing positive for the virus [8]. Recommendations highlight the importance of PA in managing Long COVID, as it has shown promise as an effective non-pharmacological therapy for mitigating persistent COVID-19 symptoms [9]. However, studies evaluating PA as a form of prevention of Long COVID are scanty. Therefore, this study aimed to investigate the relationship between PA before and after acute SARS-CoV-2 infection and the presence of Long COVID symptoms in adults.

## Methodology

We analyzed the baseline data of the Sulcovid-19, a longitudinal study that monitors the health indicators of individuals infected with COVID-19 in the city of Rio Grande, Rio Grande do Sul, Brazil. Participants should be 18 or older, have received a COVID-19 diagnosis through RT-PCR testing between December 2020 and March 2021, experienced COVID-19 symptoms, and have received medical care in Rio Grande. The Health Research Ethics Committee (CEPAS) of the Federal University of Rio Grande (FURG) (CAAE:39081120.0.0000.5324) approved the study protocol.

To identify adults who had been infected with SARS-CoV-2 contact was established with the Epidemiological Health Surveillance department of the Rio Grande. Subsequently, a list was compiled, consisting of 4,014 individuals who had tested positive for SARS-CoV-2 through RT-PCR, along with their corresponding information such as name, address, telephone number, and presence of symptoms. Following the creation of this list, inclusion and exclusion criteria were applied, resulting in 3,822 individuals being deemed eligible for the study. More information can be found in Flowchart 1 in the supplementary file.

Data collection was conducted through telephone interviews carried out by trained interviewers who underwent a rigorous selection process and received comprehensive training and qualification. The questionnaire used can be verified in the supplementary file. When necessary, home visits were offered as an alternative for face-to-face data collection. Further information regarding the study design and recruitment process can be found elsewhere [10].

We investigated a total of 19 symptoms commonly associated with Long COVID. These symptoms included headache, dyspnea (shortness of breath), dry cough, cough with phlegm, pain or discomfort when breathing, ageusia (loss of taste), anosmia (loss of smell), change in sensation (such as numbness, tingling, needling, pressure, cold/heat), fatigue, sore throat, coryza (runny nose), nasal congestion, diarrhea, nausea, arthralgia (joint pain), myalgia (muscle pain), memory loss, attention loss and alteration of the skin.

To assess Long COVID symptoms, participants were asked if they had experienced any of these symptoms after their SARS-CoV-2 infection and whether they were still experiencing them at the time of the survey. We considered a Long COVID symptom when the participant answered “yes” to both questions. Each of the 19 symptoms was analyzed as an individual outcome, and a composite variable for Long COVID was created if at least one of the symptoms was reported.

Furthermore, we categorized the symptoms into groups based on the affected body systems. These groupings included digestive symptoms (nausea and/or vomiting), musculoskeletal symptoms (muscle pain, joint pain, and/or fatigue), neurological symptoms (headache, memory loss, and/or loss of attention), respiratory symptoms (shortness of breath, dry cough, cough with phlegm, pain or discomfort in breathing, coryza, and/or nasal congestion), and sensory symptoms (ageusia, anosmia, and/or altered sensitivity). This categorization allowed for a more comprehensive analysis of the specific systems impacted by Long COVID.

We further grouped the symptoms into four broader categories, as follows: (1) musculoskeletal, neurological, and/or respiratory; (2) musculoskeletal and/or neurological; (3) musculoskeletal and/or respiratory; and (4) neurological and/or respiratory. These categories were treated as dichotomous variables, indicating whether the participant experienced at least one symptom or not.

PA was assessed based on participants self-reported frequency (days per week) and duration (time per day). Firstly, participants were asked about their PA practice in the 12 months before their SARS-CoV-2 infection. Secondly, they were asked about their PA after their infection. Those who engaged in 150 min/week or more of PA were considered active, following the WHO guidelines [11]. The independent variable was operationalized into three categories considering both timepoints, as follows: (1) remained inactive, (2) became inactive, and (3) remained active. The category “became active” was excluded due to the small number of participants (n = 14) [12].

To control for confounding the following variables were used: sex (male/female), age (18–59 years/60 years or more), income (R$ 0-1000/1001–2000/2001–4000/4001 or more in Brazilian Real), body mass index (BMI) [13] (eutrophic/low weight; overweight/obese), self-reported medical diagnosis of comorbidity (depression, hypertension, diabetes mellitus, heart problems, renal failure, respiratory problems - asthma, chronic obstructive pulmonary disease, osteoporosis, arthritis, arthrosis, or rheumatism), and hospitalization (no/yes).

All analyses were performed in Stata® 15.0. Descriptive data were presented as proportions along with their corresponding 95% confidence intervals (95%CI). We used Poisson regression to investigate the relationship between Long COVID symptoms and PA. Adjusted analyses were performed by Poisson regression with robust variance adjustment. Associations were considered statically significant when the 95% CIs did not overlap between categories.

## Results

Overall, 3,822 individuals who had tested positive for COVID-19 were eligible for the survey; after losses (631) and refusals (272), 2,919 individuals were interviewed. The interviews took place 6 to10 months after the participants infection had been diagnosed by RT-PCR testing. The prevalence of participants who experienced at least one symptom of Long COVID was 48.3% (95%CI 46.5–51.1) and most were women (58.9 95%CI 56.6–61.3) and people with a monthly income of 0-1000 (54.5 95%CI 50.7–58.3). No significant differences were found between age, ethnicity, and education level (Table 1). Regarding PA before and after infection, 71.8% (95%CI 70.1–73.4) of the participants remained inactive, 14.9% (95%CI 13.6–16.2) became inactive, and 13.3% (95% CI 12.1–14.6) remained active.

**Table 1:** Characteristics of participants with long COVID: Sulcovid-19 survey (December 2020 to March 2021). Rio Grande, Rio Grande do Sul. 2022 (n = 2919)

Variables	95% CI
*Sex*	
Female	58.9 (56.6–61.3)
Male	33.3 (30.7–36.0)
*Age*	
18–59	48.6 (46.6–50.6)
60 years or more	46.6 (42.1–51.0)
*Marital Status*	
Married/Living	48.4 (46.1–50.8)
Single / Separated / Widowed	48.0 (45.1–50.9)
*Ethnicity*	
White	48.4 (46.3–50.5)
Non-white	47.8 (43.9–51.7)
*Educational level*	
0–8 years	48.5 (44.8–52.1)
9–11 years	47.1 (44.3–49.9)
12 years or more	49.7 (46.3–53.1)
*Income*	
0–1.000.00	54.5 (50.7–58.3)
1.001-2.000.00	50.8 (47.7–53.9)
2.001-4.000.00	46.3 (42.2–50.3)
4.001.00 or more	40.9 (35.3–46.7)

Participants who remained active showed reduced prevalence of dyspnea (3.1% 95%CI 1.8–5.4), arthralgia (4.9% 95%CI 3.2–7.6), myalgia (5.2% 95%CI 3.4–7.9), headache (6.8% 95%CI 4.7–9.8), fatigue (9.1% 95%CI 6.6–12.5) and memory loss (11.7% 95%CI 8.8–15.3). Additionally, reduced prevalence on this group was observed when combining symptoms for body systems, such as respiratory (9.0% 95%CI 6.8–12.7), musculoskeletal (13.8% 95%CI 10.7–17.7) and neurological (16.8% 95%CI 13.3–20.9) and any combinations of these systems (Table 2).

**Table 2:** Prevalence of Long COVID symptoms in each PA category: Sulcovid-19 survey (December 2020 to March 2021). Rio Grande, Rio Grande do Sul, 2022 (n = 2919)

Variable	Remained inactive	Became inactive	Remained active
	% (95%CI)	% (95%CI)	% (95%CI)
Headache	11.5 (10.2–13.0)	16.9 (13.7–20.8)	6.8 (4.7–9.8)
Dyspnea	7.5 (6.4–8.7)	9.0 (6.7–12.2)	3.1 (1.8–5.4)
Dry cough	4.7 (3.8–5.7)	4.7 (3.0-7.1)	3.6 (2.2–6.1)
Phlegm	1.8 (1.3–2.5)	1.4 (0.6-3.0)	0.2 (0.0-1.8)
Pain/discomfort breathing	4.6 (3.8–5.6)	5.3 (3.6–7.9)	2.3 (1.2–4.4)
Ageusia	9.0 (7.9–10.4)	12.1 (9.3–15.6)	8.9 (6.4–12.2)
Anosmia	11.0 (9.7–12.4)	11.9 (9.1–15.4)	12.0 (9.0-15.6)
Sensitivity change	5.0 (4.1-6.0)	6.8 (4.7–9.5)	3.1 (1.8–5.4)
Fatigue	20.6 (18.9–22.4)	24.8 (20.9–29.1)	9.1 (6.6–12.5)
Sore throat	2.0 (1.5–2.7)	3.5 (2.1–5.7)	3.4 (2.0-5.8)
Coryza	3.5 (2.8–4.4)	3.7 (2.3-6.0)	2.9 (1.6–5.1)
Nasal congestion	3.0 (2.4–3.9)	3.9 (2.5–6.3)	2.9 (1.6–5.1)
Nausea / vomiting	1.0 (0.7–1.6)	1.8 (0.9–3.7)	0.5 (0.1-2.0)
Diarrhea	1.3 (0.9–1.9)	1.4 (0.6–3.1)	0.5 (0.1–2.1)
Arthralgia	9.6 (8.4–11.0)	10.0 (7.5–13.3)	4.9 (3.2–7.6)
Myalgia	10.7 (9.4–12.1)	11.4 (8.8–14.8)	5.2 (3.4–7.9)
Memory loss	18.8 (17.2–20.5)	18.6 (15.2–22.6)	11.7 (8.8–15.3)
Attention loss	14.4 (12.9–16.0)	15.6 (12.5–19.4)	9.9 (7.3–13.3)
Alteration Skin	2.5 (1.9–3.3)	5.6 (3.8–8.2)	1.8 (0.9–3.8)
Musculoskeletal	26.8 (24.9–28.7)	29.7 (25.6–34.3)	13.8 (10.7–17.7)
Neurological	27.6 (25.7–29.6)	32.2 (27.9–36.8)	16.8 (13.3–20.9)
Respiratory	16.4 (14.9–18.1)	17.9 (14.6–21.9)	9.0 (6.8–12.7)
Sensory	17.3 (15.8–19.0)	19.6 (16.1–23.7)	15.1 (11.8–19.1)
Digestive	2.3 (1.7-3.0)	2.7 (1.6–4.9)	1.4 (0.4–2.7)
Musculoskeletal + neurological + respiratory	44.0 (41.9–46.2)	46.3 (41.6–51.1)	27.3 (23.0–32.0)
Musculoskeletal + neurological	40.6 (38.5–42.8)	42.8 (38.1–47.6)	23.6 (19.6–28.2)
Musculoskeletal + respiratory	32.6 (30.6–34.6)	35.4 (31.0–40.0)	19.3 (15.7–23.6)
Neurological + respiratory	34.7 (32.7–36.8)	35.4 (31.0–40.0)	19.3 (15.7–23.6)
Long covid	49.7 (47.6–51.9)	51.9 (47.1–56.7)	36.5 (31.8–41.5)

Participants who became inactive had a higher probability of experiencing headache (PR 1.44, 95%CI 1.02; 2.03). Conversely, remaining active reduced the probability of experiencing headache by 74.0% (95%CI 0.23; 0.90). Furthermore, participants who remained active were less likely to experience fatigue (PR 0.63, 95%CI 0.41; 0.97) and at least one long COVID symptom (PR 0.80, 95%CI 0.63; 0.99) (Table 3).

**Table 3:** Crude and adjusted analysis of Long COVID symptom and PA: Sulcovid-19 survey (December 2020 to March 2021). Rio Grande, Rio Grande do Sul, 2022 (n = 2919)

Variable	Crude		Adjusted	
	Became inactive	Remained active	Became inactive	Remained active
Headache	1.47 (1.13–1.91)	0.59 (0.39–0.89)	1.44 (1.02–2.03)	0.26 (0.23–0.90)
Dyspnea	1.21 (0.86–1.72)	0.42 (0.23–0.75)	1.41 (0.87–2.30)	0.71 (0.34–1.49)
Dry cough	0.99 (0.61–1.61)	0.78 (0.44–1.36)	0.80 (0.040-1.63)	0.45 (0.16–1.27)
Phlegm	0.78 (0.33–1.85)	0.14 (0.02–1.06)	0.50 (0.11–2.13)	-
Pain/discomfort breathing	1.16 (0.73–1.82)	0.50 (0.25-1.00)	1.83 (1.01–2.32)	0.58 (0.17–1.89)
Ageusia	1.33 (0.98–1.81)	0.97 (0.68–1.40)	1.19 (0.80–1.80)	1.05 (0.65–1.68)
Anosmia	1.08 (0.80–1.47)	1.09 (0.79–1.49)	0.87 (0.57–1.32)	1.11 (0.74–1.67)
Sensitivity change	1.34 (0.89–2.03)	0.62 (0.34–1.13)	1.46 (0.82–2.59)	0.82 (0.35–1.93)
Fatigue	1.20 (0.97–1.49)	0.44 (0.31–0.63)	1.03 (0.77–1.40)	0.63 (0.41–0.97)
Sore throat	1.72 (0.96–3.11)	1.67 (0.90–3.10)	1.80 (0.80–4.06)	1.94 (0.81–4.65)
Coryza	1.06 (0.62–1.82)	0.81 (0.43–1.53)	1.03 (0.49–2.22)	1.03 (0.43–2.48)
Nasal congestion	1.30 (0.76–2.22)	0.94 (0.50–1.79)	1.09 (0.51–2.35)	1.11 (0.46–2.68)
Nausea / vomiting	1.75 (0.78–3.94)	0.49 (0.11–2.08)	1.68 (0.53–5.24)	0.73 (0.92–5.73)
Diarrhea	1.03 (0.43–2.50)	0.38 (0.09–1.62)	1.34 (0.44–4.10)	-
Arthralgia	1.04 (0.75–1.45)	0.51 (0.32–0.82)	1.35 (0.88–2.08)	0.94 (0.52–1.68)
Myalgia	1.07 (0.78–1.46)	0.49 (0.31–0.78)	1.23 (0.82–1.84)	0.77 (0.43–1.38)
Memory loss	0.99 (0.78–1.26)	0.62 (0.46–0.85)	0.92 (0.65–1.28)	0.72 (0.47–1.09)
Attention loss	1.08 (0.83–1.41)	0.69 (0.49–0.96)	1.16 (0.82–1.65)	0.85 (0.55–1.33)
Alteration Skin	2.18 (1.34–3.54)	0.71 (0.32–1.57)	1.95 (0.99–3.80)	0.90 (0.31–2.57)
Long covid	1.04 (0.90–1.21)	0.73 (0.61–0.88)	0.97 (0.80–1.17)	0.80 (0.63–0.99)

Reference: sedentary

^a^ Nausea / vomiting and Diarrhea; ^b^ Myalgia. Arthralgia and Fatigue / asthenia; ^c^ Headache. Memory loss and Attention loss; ^d^ Dyspnea. Dry cough/phlegm. Pain/discomfort breathing. Coryza. Nasal congestion; ^e^ ageusia. anosmia and sensitivity change. **Adjusted** by sex, age, BMI, education, NCDs and hospitalization

Remaining active also reduced the probability of experiencing Long COVID symptoms in the musculoskeletal (PR 0.70; 95%CI 0.49; 0.99), neurological (PR 0.61; 95%CI 0.43; 0.88), and respiratory system (PR 0.58; 95%CI 0.35; 0.96). Also, when considering the grouping of these systems together, the probability of experiencing Long COVID symptoms in those who remained active was reduced by 32–39% (Table 4).

**Table 4:** Crude and adjusted analysis of clustered Long COVID symptoms and PA: Sulcovid-19 survey (December 2020 to March 2021). Rio Grande, Rio Grande do Sul, 2022 (n = 2919)

Variable	Crude		Adjusted	
	Became inactive	Remained active	Became inactive	Remained active
Musculoskeletal	1.11 (0.91–1.35)	**0.52 (0.39–0.68)**	0.95 (0.73–1.26)	**0.70 (0.49–0.99)**
Neurological	1.17 (0.97–1.40)	**0.61 (0.47–0.79)**	1.15 (0.90–1.47)	**0.61 (0.43–0.88)**
Respiratory	1.09 (0.85–1.40)	**0.57 (0.40–0.80)**	1.14 (0.81–1.60)	**0.58 (0.35–0.96)**
Sensory	1.13 (0.89–1.43)	0.87 (0.66–1.15)	1.03 (0.76–1.42)	0.95 (0.66–1.35)
Digestive	1.21 (0.64–2.27)	0.45 (0.17–1.24)	1.59 (0.71–3.54)	0.27 (0.04–2.04)
Musculoskeletal + neurological + respiratory	1.05 (0.90–1.23)	**0.62 (0.51–0.76)**	0.96 (0.78–1.18)	**0.64 (0.49–0.84)**
Musculoskeletal + neurological	1.05 (0.90–1.24)	**0.58 (0.47–0.72)**	0.95 (0.77–1.18)	**0.62 (0.46–0.83)**
Musculoskeletal + respiratory	1.08 (0.91–1.29)	**0.59 (0.46–0.75)**	0.95 (0.74–1.21)	**0.68 (0.50–0.94)**
Neurological + respiratory	1.14 (0.96–1.35)	**0.63 (0.50–0.78)**	1.10 (0.88–1.37)	**0.61 (0.44–0.84)**

Reference: Remained inactive

## Discussion

Almost half of participants experienced at least one Long COVID symptom, and only 13.3% remained active. We revealed that remaining active reduced the probability of experience Long COVID symptoms, and specific symptoms such as fatigue. Also, continuous practice of PA reduced the probability of experiencing symptoms on respiratory, musculoskeletal and neurological systems.

One of the reasons behind the association between continuous PA and respiratory symptoms is the affinity of the SARS-Cov-2 virus with pericytes, leading to their elimination [14]. Consequently, individuals infected with COVID-19 tend to have lower cardiorespiratory fitness compared to healthy individuals. This is due to reduced peripheral oxygen extraction rate, increased venous saturation [15], and a resultant decrease in exercise tolerance [14]. Engaging in PA is crucial since enhances mitochondrial activity and improves oxygen uptake, thereby preserving energy production during cellular respiration [16]. Fatigue, which is reported as one of the primary symptoms following SARS-CoV-2, with a prevalence of 47.0% [17, 18], can be prevented by engaging in PA, as supported by our data.

Myalgia, the third most prevalent symptom observed in individuals following acute infection, has a prevalence of 25.0% [17]. This can be attributed to the affinity of the SARS-CoV-2 for angiotensin-converting enzyme 2 (ACE2), which is present in muscle tissue, leasing to direct damage to muscle tissue [19, 20]. Regular PA provides benefits to the osteomuscular system by strengthening the immune system, which aids in the detection and elimination of infected cells, particularly in tissues with ACE2 expression, such as muscle tissues [21, 22].

Neurological symptoms, such as memory and attention loss, can be attributed to two main factors. Firstly, possible vascular, hemorrhagic, and ischemic injuries, as well as microglial activation in the white matter, T-cell invasion, and regional neuronophagy in the locus coeruleus, which is responsible for brain functions such as attention and memory [23–25]. Secondly, there may be a psychosomatic effect due to the trauma of contracting the virus [26]. Individuals who have recovered from COVID-19 often report feeling unlike themselves, experiencing short-term memory loss, confusion, difficulty concentrating, and a general sense of being different compared to before the infection [26, 27].

Engaging in continuous PA has beneficial impact on neurogenesis and synaptic plasticity associated, which can help alleviate minor neurological complications. Notably, PA promotes the production of astrocytes, which are central nervous system cells responsible for maintaining brain homeostasis [28, 29]. Our data supports the potential advantages of maintaining regular physical activity in the context of Long COVID. These findings are crucial as approximately 50% of individuals affected by COVID-19 may experience the syndrome [5].

The etiology of headache as a residual symptom is complex and multifactorial, involving direct infection effects, cerebrovascular disease (including hypercoagulation), physiological impairments (e.g., hypoxia), medication side effects, and social aspects related to having a life-threatening illness [30]. Continuous PA appears to play a role maintaining brain health by regulating neurotrophic factors and anti-inflammatory cytokines [31]. Thus, the significance of our findings underscores the potential benefits of continuous PA in the prevention of Long COVID symptoms [32].

Despite the challenges posed by the closure of exercise spaces due to social distancing requirements [33], coupled with the difficulties faced by individuals with Long COVID in resuming PA due to their symptoms, our findings indicate that regular PA should be considered as a non-pharmacological strategy to reduce the likelihood of developing Long COVID. In other words, PA promoting policies should be included in first response action in pandemic scenarios, such as the COVID-19. It is important to highlight the magnitude of protection that PA can provide for the various symptoms, considering that the estimated overall prevalence of Long COVID in non-hospitalized patients can reach values of 34.0% (95% CI, 25.0–46.0) [4].

The limitations of our study should be listed. Firstly, we did not assess asymptomatic individuals during the acute phase of SARS-CoV-2 infection. Consequently, sample bias cannot be discarded. Secondly, PA was assessed retrospectively, and memory bias might be a concern. Thirdly, the questionnaire focused on the 19 most prevalent symptoms reported in the literature. Even though there are more than 200 Long COVID symptoms reported, we were unable to cover all of them in the questionnaire due to interview restraints [5]. Fourthly, we only analyzed individuals who survived COVID-19, and survival bias cannot be discarded. However, our sample consisted of individuals with COVID-19 who were diagnosed using the gold standard test (RT-PCR), and we achieved a high response rate (> 75%). Furthermore, different from other studies, we evaluated different PA levels in individuals who were not hospitalized.

## Conclusion

The continuous PA practice showed a protective effect against Long COVID symptoms, particularly those affecting the musculoskeletal, neurological and respiratory systems. These findings enhance our understanding of the association between PA and Long COVID symptoms, providing a theoretical basis for healthcare managers to promote PA during pandemic scenarios. To facilitate continuous PA strategies, it is recommended to encourage home-based exercises and utilize digital platforms that provide guidance from exercise science professionals. Implementing these initiatives aims to assist individuals in sustaining an active lifestyle despite the challenges posed by the pandemic scenario.

### Electronic supplementary material

Below is the link to the electronic supplementary material.

Supplementary Material 1

Supplementary Material 2

## Data Availability

Datasets used and/or analyzed during the current study are available from the corresponding author upon reasonable request.

## Abbreviations

PA: Physical activity
CEPAS: Health Research Ethics Committee
FURG: Federal University of Rio Grande
RT-PCR: Reverse transcription polymerase chain reaction
