# Supplementary material for: Physical activity status prevents symptoms of long covid: Sulcovid-19 survey
Source: BMC Sports Sci Med Rehabil. 2023 Dec 14;15:170. doi: 10.1186/s13102-023-00782-5 (PMC10722691; doi:10.1186/s13102-023-00782-5)
Supplement: Supplementary file 1 — Supplementary Material 1 [file 13102_2023_782_MOESM1_ESM.pdf]

SULCOVID-19 Questionnaire

interview code:	code _ _ _ _ _	
BLOCK A - GENERAL DATA<br><br>Good morning/afternoon/evening. My name is XXXX. I am a researcher for the SulCOVID study at FURG. I would like to speak with Mr. XXXX. Our research aims to evaluate the people's health after infection with COVID-19 between December/2020 and March/2021.<br>Your responses will be kept confidential and your identity will be preserved. The interview lasts approx. 20 minutes and for your safety, it will be recorded.<br>We inform you that this research is regulated by the Research Ethics Committee of FURG.		
Can we start the interview?	Yes No	entre
	If no Can we contact you at another time?<br>Yes No	acontato
	If Yes, what is the best day of the week and time to contact you?<br>☐ Monday (morning)<br>☐ Second(afternoon)<br>☐ Second(night)<br>☐ Tuesday (morning)<br>☐ Tuesday (afternoon)<br>☐ Tuesday (night)<br>☐ Wednesday (morning)<br>☐ Fourth(afternoon)<br>☐ Fourth(night)<br>☐ Thursday (morning)<br>☐ Fifth(afternoon)<br>☐ Fifth(night)<br>☐ Friday (morning)<br>☐ Friday (afternoon)<br>☐ Friday night<br>☐ Saturday (morning)<br>☐ Saturday(afternoon)<br>☐ Saturday(night)<br>☐ Sunday (morning)<br>☐ Sunday (afternoon)<br>☐ Sunday (night)	aentre2
	If no - Do you authorize us to come by your home to carry out the interview?	aentre3
	If Yes Can you confirm your address?<br>If No Finish the questionnaire	aentre4

BLOCK A - GENERAL DATA

What's your full name?		anome
Do you have any other phone number?		atel
What is your WhatsApp?	(0) I don't have<br>(1) same number<br>(2) another number	awhats
What is the number?		awhatsn
Sex	(0) Masculine<br>(1) Feminine<br>(888) don't know or didn't want to answer	asexo
How old are you (in years)?		aidade
What is your birth date?	_ _ / _ _ / _ _ _ _	adn

What is your color/race?	(0) White<br>(1) black<br>(2) Yellow<br>(3) brown<br>(4) Indigenous<br>(888) Don't know or didn't want to answer	acor
Do you know how to read and write?	(0) No > SKIP TO What's your height?<br>(1) Yes<br>(888) Don't know or didn't want to answer	aler
To what degree did you study?	(0) Never studied. > SKIP TO What's your height?<br>(1) 1st grade: elementary school, that is, from the first to the eighth grade.<br>(2) 2nd GRADE: high school, that is, the first or third year.<br>(3) 3rd DEGREE: higher education, that is, college.<br>(888) Don't know or didn't want to answer	agrau
Up to what grade did you study? IF YES TO 1st GRADE QUESTION	1st Grade (* Consider completed grades):<br>(0) Preschool (Kindergarten)<br>(1) 1st grade of Elementary School<br>(2) 2nd grade of Elementary School<br>(3) 3rd grade of Elementary School<br>(4) 4th grade of Elementary School<br>(5) 5th grade of elementary school<br>(6) 6th grade of elementary school<br>(7) 7th grade of Elementary School<br>(8) 8th grade of elementary school (888) does not know or did not want to answer	a1grau
Up to what grade did you study? IF YES TO STUDIED HIGH SCHOOL	2nd Grade (*Consider completed grades):<br>(0) 1st year of high school<br>(1) 2nd year of high school<br>(2) 3rd year of high school<br>(888) don't know or didn't want to answer	a2grau
Up to what grade did you study? IF YOU ANSWER YES TO STUDIED HIGH SCHOOL	(0) Currently in higher education<br>(1) Completed Higher Education<br>(888) don't know or didn't want to answer	a3grau
What's your height?	cm Ex: 1.72 m = 172 cm<br>(888) don't know or didn't want to answer	aaltura
What's your weight?	kg Ex: 74.6 kg = 74.6<br>(888) don't know or didn't want to answer	apeso
**BLOCK B2 - COVID-19 INFECTION**		
Which of the following symptoms did you have after being infected with COVID-19?		
Headache	(0) No<br>(1) Yes<br>(888) don't know or didn't want to answer	bdorc
IF YES > Currently, do you still experience this symptom?	(0) No<br>(1) Yes<br>(888) don't know or didn't want to answer	bdorcp
Shortness of breathe	(0) No<br>(1) Yes<br>(888) don't know or didn't want to answer	bfalta

IF YES > Currently, do you still experience this symptom?	(0) No<br>(1) Yes<br>(888) don't know or didn't want to answer	bfaltap
Dry cough	(0) No<br>(1) Yes<br>(888) don't know or didn't want to answer	btosse
IF YES > Currently, do you still experience this symptom?	(0) No<br>(1) Yes<br>(888) don't know or didn't want to answer	btossep
Cough with phlegm	(0) No<br>(1) Yes<br>(888) don't know or didn't want to answer	bcatarro
IF YES > Currently, do you still experience this symptom?	(0) No<br>(1) Yes<br>(888) don't know or didn't want to answer	bcatarrop
Pain/discomfort breathing	(0) No<br>(1) Yes<br>(888) don't know or didn't want to answer	bdorresp
IF YES > Currently, do you still experience this symptom?	(0) No<br>(1) Yes<br>(888) don't know or didn't want to answer	bdorrespp
Loss of taste	(0) No<br>(1) Yes<br>(888) don't know or didn't want to answer	bpaladar
IF YES > Currently, do you still experience this symptom?	(0) No<br>(1) Yes<br>(888) don't know or didn't want to answer	bpaladarp
Loss of smell	(0) No<br>(1) Yes<br>(888) don't know or didn't want to answer	bolfato
IF YES > Currently, do you still experience this symptom?	(0) No<br>(1) Yes<br>(888) don't know or didn't want to answer	bolfatop
Change in sensitivity (feelings of falling asleep, tingling, pins and needles, pressure, cold/heat)	(0) No<br>(1) Yes<br>(888) don't know or didn't want to answer	bsensi
IF YES > Currently, do you still experience this symptom?	(0) No<br>(1) Yes<br>(888) don't know or didn't want to answer	bsensip
Fatigue	(0) No<br>(1) Yes<br>(888) don't know or didn't want to answer	bcansaco
IF YES > Currently, do you still experience this symptom?	(0) No<br>(1) Yes<br>(888) don't know or didn't want to answer	bcansacop
Sore throat	(0) No<br>(1) Yes<br>(888) don't know or didn't want to answer	bdorgar
IF YES > Currently, do you still experience this symptom?	(0) No<br>(1) Yes<br>(888) don't know or didn't want to answer	bdorgarp
Runny nose (runny nose, explain with popular name)	(0) No<br>(1) Yes<br>(888) don't know or didn't want to answer	bcoriza
IF YES > Currently, do you still experience this symptom?	(0) No<br>(1) Yes<br>(888) don't know or didn't want to answer	bcorizap
Nasal congestion (stuffy nose)	(0) No<br>(1) Yes<br>(888) don't know or didn't want to answer	bnasal
IF YES > Currently, do you still experience this symptom?	(0) No<br>(1) Yes<br>(888) don't know or didn't want to answer	bnasalp
Nausea or vomiting	(0) No<br>(1) Yes<br>(888) don't know or didn't want to answer	bnausea

IF YES > Currently, do you still experience this symptom?	(0) No<br>(1) Yes<br>(888) don't know or didn't want to answer	bnauseap
Diarrhea	(0) No<br>(1) Yes<br>(888) don't know or didn't want to answer	bdiarreia

IF YES > Currently, do you still experience this symptom?	(0) No<br>(1) Yes<br>(888) don't know or didn't want to answer	bdiarreiap
Joint pain (joints)	(0) No<br>(1) Yes<br>(888) don't know or didn't want to answer	bdorart
IF YES > Currently, do you still experience this symptom?	(0) No<br>(1) Yes<br>(888) don't know or didn't want to answer	bdorartp
Muscle pain	(0) No<br>(1) Yes<br>(888) don't know or didn't want to answer	bdormusc
IF YES > Currently, do you still experience this symptom?	(0) No<br>(1) Yes<br>(888) don't know or didn't want to answer	bdormuscp
Memory loss	(0) No<br>(1) Yes<br>(888) don't know or didn't want to answer	bmem
IF YES > Currently, do you still experience this symptom?	(0) No<br>(1) Yes<br>(888) don't know or didn't want to answer	bmemp
Loss of attention	(0) No<br>(1) Yes<br>(888) don't know or didn't want to answer	baten
IF YES > Currently, do you still experience this symptom?	(0) No<br>(1) Yes<br>(888) don't know or didn't want to answer	batenp
Skin changes (in the skin)	(0) No<br>(1) Yes<br>(888) don't know or didn't want to answer	bpele
IF YES > Currently, do you still experience this symptom?	(0) No<br>(1) Yes<br>(888) don't know or didn't want to answer	bpelep
Other?	(0) No<br>(1) Yes<br>(888) don't know or didn't want to answer	bout
IF YES > Which?		bout2
IF YES > Currently, do you still experience this symptom?	(0) No<br>(1) Yes<br>(888) don't know or didn't want to answer	boutp
**BLOCK F - NOW WE WILL TALK ABOUT YOUR PRACTICE OF PHYSICAL EXERCISE**		
During the 12 months before your COVID-19 infection, on average, how many days a week did you practice some type of physical exercise or sport? (do not consider physiotherapy)	(0) no day<br>(1) 1 day<br>(2) 2 days<br>(3) 3 days<br>(4) 4 days<br>(5) 5 days<br>(6) 6 days<br>(7) 7 days<br>(888) Don't know or didn't want to answer	eef
IF FROM 1 TO 7 DAYS >On average, how many minutes did this activity last each day?	_ _ _ _ minutes<br>(888) Don't know or didn't want to answer	eeft
**AFTER** your COVID-19 infection, on average, how many days a week do you exercise or play sports?	(0) no day<br>(1) 1 day<br>(2) 2 days<br>(3) 3 days<br>(4) 4 days<br>(5) 5 days<br>(6) 6 days<br>(7) 7 days<br>(888) Don't know or didn't want to answer	eefp
IF FROM 1 TO 7 DAYS >On average, how many minutes did this activity last each day?	_ _ _ _ minutes<br>(888) Don't know or didn't want to answer	eeftp

BLOCK G – NOW LET'S TALK ABOUT YOUR HEALTH		
At some point in your life, has any doctor or psychologist ever told you that you had depression?	(0) No<br>(1) Yes<br>(888) Don't know or didn't want to answer	gdepre
After your COVID-19 infection so far, has any doctor or psychologist told you that you have depression?	(0) No<br>(1) Yes<br>(888) Don't know or didn't want to answer	gdeprep
Has a doctor or psychologist ever told you that you have another mental or emotional illness, such as anxiety, schizophrenia, bipolar disorder, or OCD? (obsessive compulsive disorder)?	(0) No<br>(1) Yes<br>(888) Don't know or didn't want to answer	gansi
In the past two weeks, how many days have you thought about hurting yourself in some way or thinking that you would be better off dead?	(0) no day<br>(1) Less than a week<br>(2) a week or more<br>(3) Almost everyday<br>(888) don't know or didn't want to answer	gsui
At some point in your life, has a doctor ever told you that you have:<br><br>Breathing problems (Asthma, Bronchitis, Emphysema or COPD)?	(0) No<br>(1) Yes<br>(888) Don't know or didn't want to answer	gresp
Osteoporosis or weak bones?	(0) No<br>(1) Yes<br>(888) Don't know or didn't want to answer	gost
Arthritis, arthrosis, or rheumatism?	(0) No<br>(1) Yes<br>(888) Don't know or didn't want to answer	greu
Hypertension (i.e., high blood pressure),<br>even if controlled?	(0) No<br>(1) Yes<br>(888) Don't know or didn't want to answer	ghas
Diabetes, even if controlled?	(0) No<br>(1) Yes<br>(888) Don't know or didn't want to answer	gdia
Heart problem? (e.g., heart failure)?	(0) No<br>(1) Yes<br>(888) Don't know or didn't want to answer	gcor
Eye problems (e.g., glaucoma, cataracts, retinopathy)?	(0) No<br>(1) Yes<br>(888) Don't know or didn't want to answer	goals
Cancer?	(0) No<br>(1) Yes<br>(888) Don't know or didn't want to answer	gcancer
Urinary or fecal incontinence?	(0) No<br>(1) Yes<br>(888) Don't know or didn't want to answer	gincont
Any other long-term illness (longer than 6 months)?	(0) No<br>(1) Yes<br>(888) Don't know or didn't want to answer	gdoen
**BLOCK P - SOCIOECONOMIC DATA**<br>**NOW LET'S TALK ABOUT SOME FEATURES OF YOUR HOUSE**		

How much did you received in the last month (including salary, pension, time off compensation)?	(0) Has no income<br>(1) Less than BRL 500.00<br>(2) From BRL 500.00 to BRL 1,000.00<br>(3) From BRL 1,001.00 to BRL 2,000.00<br>(4) From BRL 2,001.00 to BRL 4,000.00<br>(5) From BRL 4,001.00 to BRL 6,000.00<br>(6) From BRL 6,001.00 to BRL 8,000.00<br>(7) From BRL 8,001.00 to BRL 10,000.00<br>(8) From BRL 10,001.00 to BRL 20,000.00<br>(9) More than BRL 20,000.00<br>(888) Don't know or didn't want to answer	prenda
**THANK YOU AND CLOSE THE QUESTIONNAIRE**		
