# Supplementary material for: Physical activity status prevents symptoms of long covid: Sulcovid-19 survey
Source: BMC Sports Sci Med Rehabil. 2023 Dec 14;15:170. doi: 10.1186/s13102-023-00782-5 (PMC10722691; doi:10.1186/s13102-023-00782-5)
Supplement: Supplementary file 2 — Supplementary Material 2 [file 13102_2023_782_MOESM2_ESM.pdf]

Flowchart 1. Recruitment and exclusion process of individuals infected by SARS-CoV-2. Sulcovid-19 Survey.

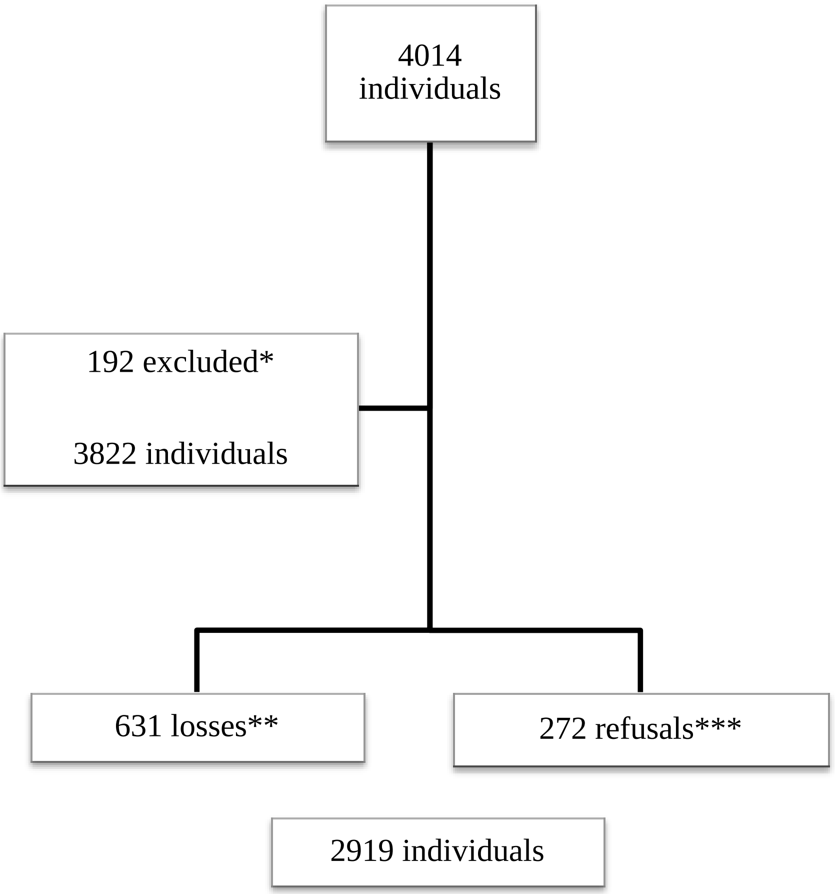

*Excluded due to lack of information such as address and telephone.

**Individuals who did not respond to the three telephone contacts and who were not found at home during home visits were considered losses. Individuals whose address could not be found were also considered lost.

*** Individuals' refusals included, among others, lack of time and interest in talking about their infection.
